# Civility

**Published:** 1857-04

**Authors:** 


### CIVILITY

COSTS nothing, and considering it pays its way so handsomely in all companies, to say nothing of occasional chance advantages, it is a marvel that it is not more common—that it is not a universal virtue.

Within a very few years, a couple of gentlemen—one of whom was a foreigner—visited the various locomotive workshops of Philadelphia. They called at the most prominent one first, stated their wishes to look through the establishment, and make some inquiries of a more specific character. They were shown through the premises in a very indifferent manner, and no special pains were taken to give them any information beyond what their own inquiries drew forth. The same results followed their visits to the several larger establishments. By some means, they were induced to call on one of a third or fourth rate character. The owner was himself a workman, of limited means; but on the application of the strangers, his natural urbanity of manner prompted him not only to show all that he had, but to enter into a detailed explanation of the working of his establishment, and of the very superior manner in which he could conduct his factory if additional facilities of capital were afforded him. The gentlemen left him, not only favorably impressed towards him, but with the feeling that he thoroughly understood his business.

Within a year he was surprised with an invitation to visit St. Petersburg. The result was, his locomotive establishment was removed there bodily. It was the agent of the Czar who had called on him, in company with an American citizen. He has recently returned, having accumulated a large fortune, and still receives from his Russian workshops about a hundred thousand dollars a year. He invests his money in real estate, and has already laid the foundation for the largest fortune of any private individual in Philadelphia; and all the result of civility to a couple of strangers.

